# Screening of Dietary Flavonoids for Synergistic α-Glucosidase Inhibition with 1-Deoxynojirimycin and Elucidation of the Underlying Molecular Mechanism

**DOI:** 10.3390/molecules31162907

**Published:** 2026-08-20

**Authors:** Lin Wang, Jun Liu, Yonghong Zhao, Zhongshan Xiao, Lei Zeng, Zhuming Liu, Wei Wang, Baogang Wang, Jinping Wang

**Affiliations:** 1 School of Pharmacy, Xinyang Agriculture and Forestry University, Xinyang 464000, China; 2021260007@xyafu.edu.cn (L.W.);; 2 Nanjing Institute for Food and Drug Control, Nanjing 211198, China; 3 Department of Pharmacy, Puyang Medical College, Puyang 475000, China; 4 College of Food Science and Engineering, Xinyang Agriculture and Forestry University, Xinyang 464000, China

**Keywords:** α-Glucosidase, DNJ, (+)-catechin, synergistic inhibition, allosteric modulation

## Abstract

1-Deoxynojirimycin (DNJ), a well-characterized α-glucosidase inhibitor, remains an important target for dose-reduction and formulation strategies. In the present study, we evaluated the individual and combined α-glucosidase inhibitory activities of nine dietary flavonoids with DNJ, quantified synergistic effects using the combination index (CI) method, and elucidated the molecular mechanism through integrated enzyme kinetics, multi-spectroscopic techniques and molecular docking. (+)-Catechin exhibited the strongest inhibitory activity (IC_50_ = 33.7 ± 2.7 μM), and its combination with DNJ produced synergistic inhibition across all doses (CI < 0.7). Kinetic analysis confirmed that DNJ acted as a competitive inhibitor, while (+)-catechin functioned as a non-competitive inhibitor. Fluorescence quenching assays revealed that (+)-catechin pre-incubation increased the binding affinity of DNJ to α-glucosidase by 393%. Circular dichroism spectroscopy showed that (+)-catechin induced a marked β-sheet-to-α-helix conformational conversion, and co-incubation of both inhibitors produced more secondary structural changes than either inhibitor alone. Molecular docking further confirmed their distinct binding sites. These findings demonstrate that (+)-catechin synergistically potentiates DNJ activity through allosteric conformational modulation, providing an experimental basis for optimizing DNJ-containing formulations.

## 1. Introduction

With rising living standards and shifting dietary patterns, the global prevalence of diabetes mellitus is increasing rapidly, making it one of the most prevalent chronic metabolic diseases globally [1,2]. Postprandial hyperglycemia is a defining feature of diabetes pathogenesis, particularly in T2DM [3]. Therefore, effective control of postprandial blood glucose is critical for both the prevention and management of the disease. α-Glucosidase is a key hydrolase distributed on the brush border membrane of the small intestine that mediates the hydrolysis of starch and oligosaccharides into absorbable monosaccharides and serves as a rate-limiting enzyme in carbohydrate digestion. α-Glucosidase inhibitors (AGIs) delay carbohydrate hydrolysis and absorption, effectively reducing postprandial blood glucose peaks, and are currently first-line agents for the clinical management of T2DM [4]. The approved AGIs include acarbose [5], voglibose [6], and miglitol [7]. However, these agents are frequently associated with gastrointestinal adverse effects such as bloating, diarrhea, and flatulence, which limit their clinical utility and patient adherence. This has driven intensive research efforts to identify natural AGIs with high efficacy and low toxicity. 1-Deoxynojirimycin (1-DNJ) is a sugar-mimicking piperidine alkaloid first isolated and identified from mulberry root bark [8]. Among mulberry alkaloids, 1-DNJ is the principal α-glucosidase inhibitor, and its reported activity in postprandial glucose management is primarily attributed to inhibition of this enzyme, which delays carbohydrate digestion and absorption [9]. Although 1-DNJ also exhibits other biological activities, α-glucosidase inhibition remains the activity most directly linked to its antidiabetic relevance. Pharmacokinetic studies indicate that DNJ is rapidly absorbed and excreted, and that its oral bioavailability may limit its therapeutic application [10,11,12]. Therefore, combining DNJ with other bioactive compounds represents a rational strategy for improving its dose efficiency and formulation potential.

Flavonoids are the most abundant polyphenols in plants and important bioactive components in the human diet, with a broad spectrum of biological activities such as α-glucosidase inhibition, antioxidant, and anti-inflammatory effects [13,14]. Recent studies have revealed that certain flavonoids possess intrinsic α-glucosidase inhibitory activity and may act synergistically with DNJ, enabling a reduced DNJ dosage while maintaining overall inhibitory efficacy. For example, the combination of DNJ and morin ameliorates insulin resistance and inhibits adipogenesis [15], and the combination of total alkaloids from *Feculae bombycis* with catechin or quercetin exerts synergistic inhibitory effects on yeast α-glucosidase [16]. However, current research on flavonoid–DNJ combinations remain largely at the descriptive level: most studies only compare simple inhibition rates, lack quantitative evaluation of synergistic effects using the combination index (CI) method, and rarely involve systematic enzyme kinetic analysis and molecular mechanism exploration. It remains unclear whether the interaction is additive or synergistic, and the underlying molecular basis is still poorly understood.

**Figure 1:**
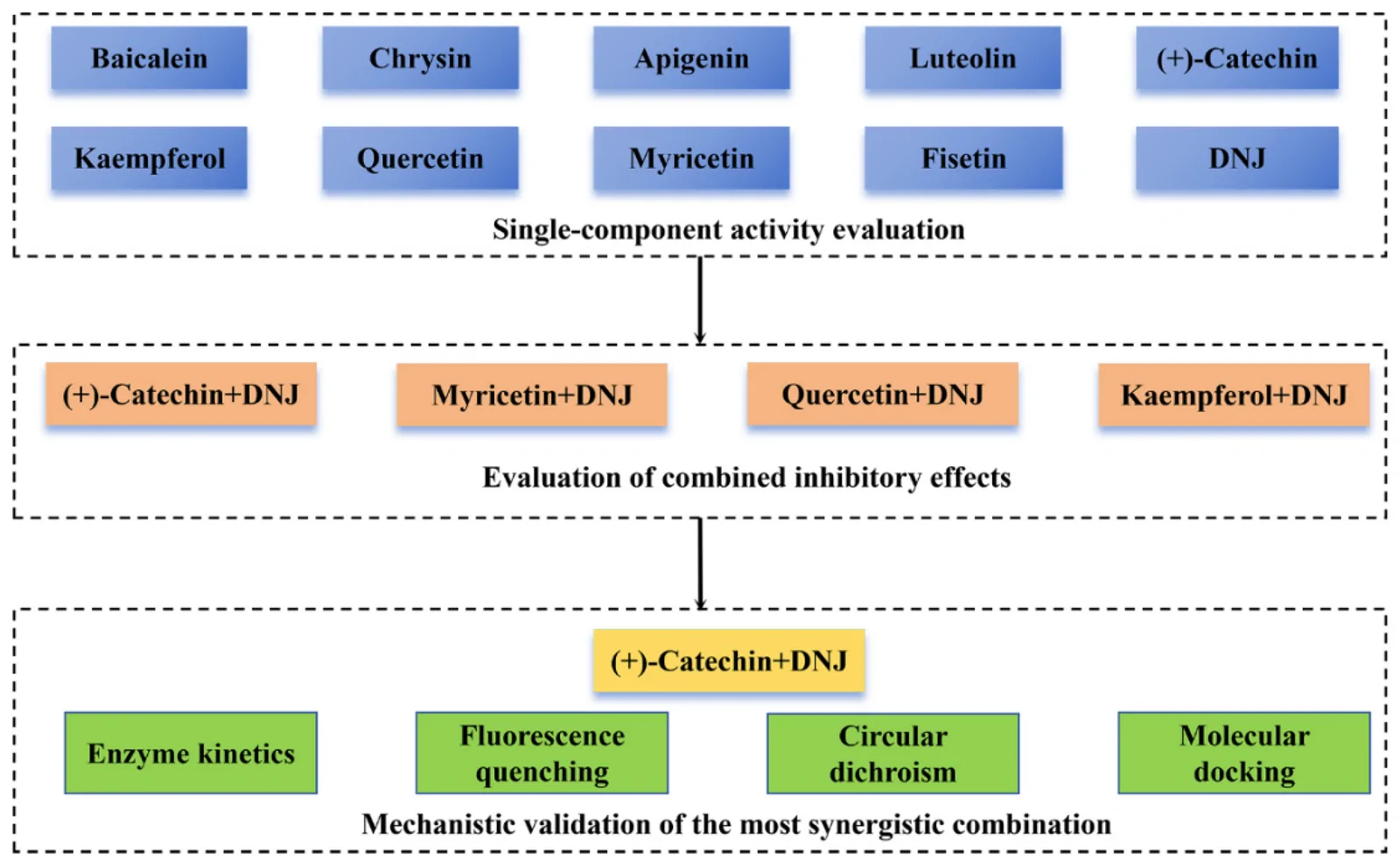
A schematic diagram of this study.

In the present study, we evaluated the individual and combined α-glucosidase inhibitory activities of nine dietary flavonoids and screened for combinations exhibiting significant synergistic effects with DNJ. Building on these results, the molecular mechanism of the observed synergistic inhibition was systematically investigated using enzyme inhibition kinetics, fluorescence spectroscopy, circular dichroism (CD) spectroscopy, and molecular docking. These flavonoids were selected because they differ systematically in the number and position of hydroxyl groups on their A-, B-, and C-rings, allowing structure–activity relationships to be examined for both individual inhibition and synergistic interaction with DNJ. All compounds were commercially sourced analytical standards; no compounds were isolated from plant material in this study. The aim was to reveal the structural basis and allosteric modulation mechanism of flavonoid–DNJ synergistic inhibition, and to provide a mechanistic foundation for the rational design of optimized DNJ-based formulations. A schematic diagram is presented in Figure 1.

## 2. Results and Discussion

### 2.1. Individual α-Glucosidase Inhibitory Activity of Flavonoids

**Figure 2:**
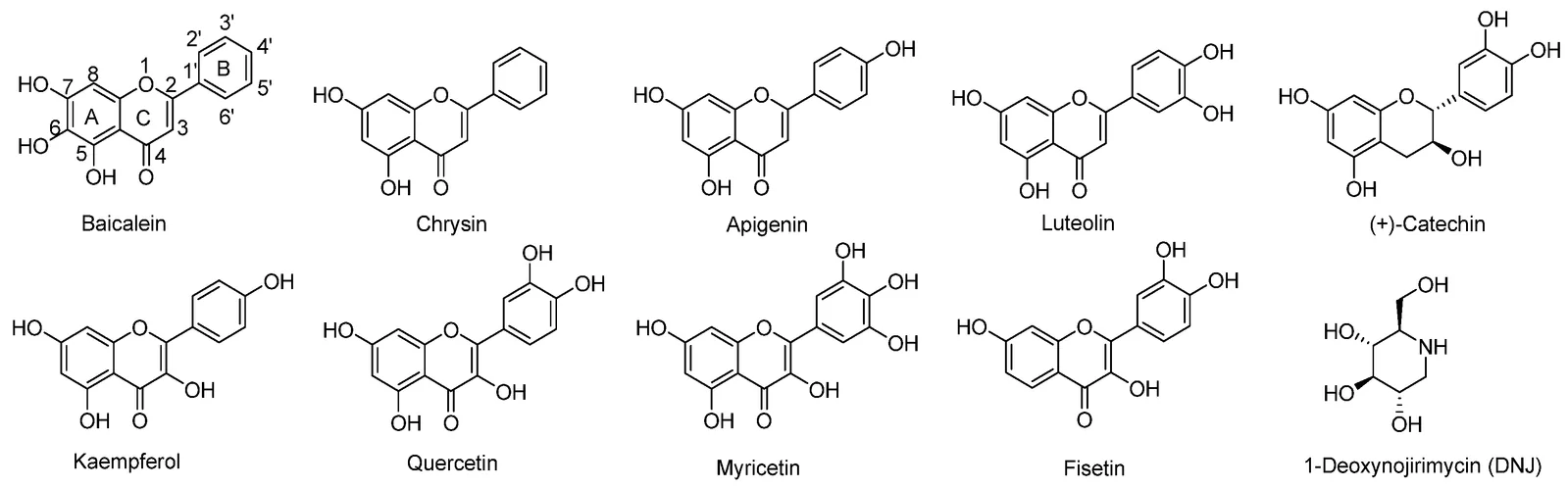
Structures of the tested flavonoids and DNJ.

The α-glucosidase inhibitory activities of nine commercially available flavonoids (Figure 2) bearing distinct hydroxyl substitutions on their A-, B-, and C-rings were evaluated. DNJ was used as a positive control. As shown in Table 1, the IC_50_ value of DNJ was 155.5 ± 17.2 μM. (+)-Catechin and quercetin were the most potent inhibitors, with IC_50_ values of 33.7 ± 2.7 μM and 160.0 ± 5.1 μM, respectively. Kaempferol (163.2 ± 10.0 μM) and myricetin (395.0 ± 14.9 μM) showed weaker activity than (+)-catechin, while all other tested flavonoids had IC_50_ values > 400 μM.

Structure–activity relationship (SAR) analysis revealed clear trends. Quercetin was more active than kaempferol or myricetin, with kaempferol being more active than myricetin. Hydroxylation at the 4′-position of the B-ring of chrysin, giving apigenin, increased the activity. These results indicated that the presence of the −OH groups at the 3′- and 4′-positions was more relevant than that at the 5′-position of the B-ring. Comparison of kaempferol and apigenin further showed that a 3-OH group in the C-ring enhanced α-glucosidase inhibition. Xu et al. [17] corroborated these SAR trends via AutoDock (version 1.2.6) molecular docking, demonstrating that the 3′- and 4′-OH groups of the B-ring, together with the 3-OH in the C-ring, are essential for activity. Nicolle et al. [18] and Silva et al. [19] suggested that the 3-OH in the C-ring may help flavonoids adopt a suitable binding orientation in the active pocket of yeast α-glucosidase. We further found that the 5-OH in the A-ring was important for α-glucosidase inhibition, since quercetin was more effective than fisetin. Wang et al. [20] showed that acetylation of the 5-position of the A-ring of chrysin nearly abolished inhibitory activity. Consistently with the critical role of hydrogen bonding in enzyme–inhibitor interactions, Zheng et al. [21] demonstrated that the A-ring 5-OH group acts as a hydrogen bond donor, thereby enhancing flavonoid inhibitory activity. (+)-Catechin exhibited the strongest α-glucosidase inhibition among the tested flavonoids and the positive control DNJ. This result is consistent with the view that the binding mode and structural features of catechins strongly influence their α-glucosidase inhibitory activity [22].

### 2.2. Combined Inhibitory Effect of Flavonoids and DNJ on α-Glucosidase

**Figure 3:**
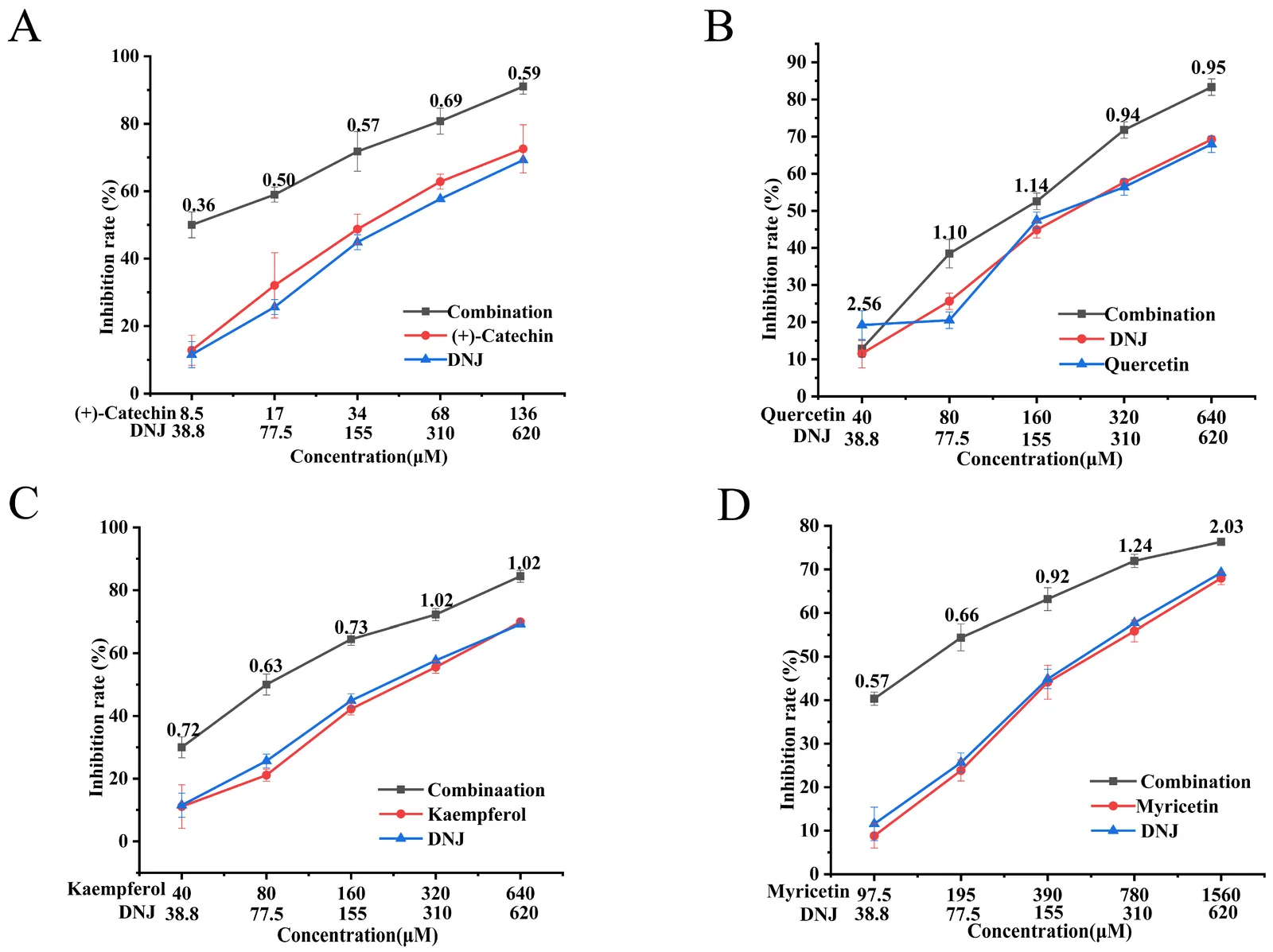
Inhibitory effects of DNJ–flavonoid combinations against α-glucosidase. Combinations of DNJ with (+)-catechin (**A**), quercetin (**B**), kaempferol (**C**) and myricetin (**D**). CI values marked above data points were calculated using CompuSyn software 1.4. According to the established criterion, CI values below 0.9, between 0.9 and 1.1, and above 1.1 correspond to synergistic, additive, and antagonistic interactions, respectively. All experiments were performed in triplicate.

Having established the individual inhibitory activities of the nine flavonoids, we selected the four most potent compounds, (+)-catechin, quercetin, kaempferol, and myricetin (IC_50_ < 400 μM), for combination studies with DNJ. The combination of (+)-catechin and DNJ inhibited α-glucosidase more potently than either compound alone: coadministration of 8.5 μM (+)-catechin and 38.8 μM DNJ achieved 50.0% inhibition, exceeding that produced by twice the concentration of either agent alone (17 μM (+)-catechin or 77.5 μM DNJ) (Figure 3A). CI values remained below 0.7 across all tested dose points, indicating strong synergy. In contrast, kaempferol and myricetin combined with DNJ showed moderate synergy at low doses (CI: 0.6–0.9) but became additive or antagonistic at high doses (CI > 1.0) (Figure 3C,D). Quercetin produced antagonism at low doses and additivity at high doses (Figure 3B).

A distinct structure–activity relationship emerged from the combination experiments. At low doses, (+)-catechin, kaempferol, and myricetin all synergistically inhibited α-glucosidase with DNJ, but only (+)-catechin retained synergy at high doses. Individual inhibitory potency did not predict synergistic behavior: quercetin, the second most potent inhibitor alone, exhibited antagonism with DNJ at low doses, whereas (+)-catechin was the strongest inhibitor in both assays. This dissociation suggests that the structural determinants of synergy differ from those governing active-site binding, and that individual potency and synergy should be assessed independently when screening for combination partners.

### 2.3. Analysis of Inhibition Types

**Figure 4:**
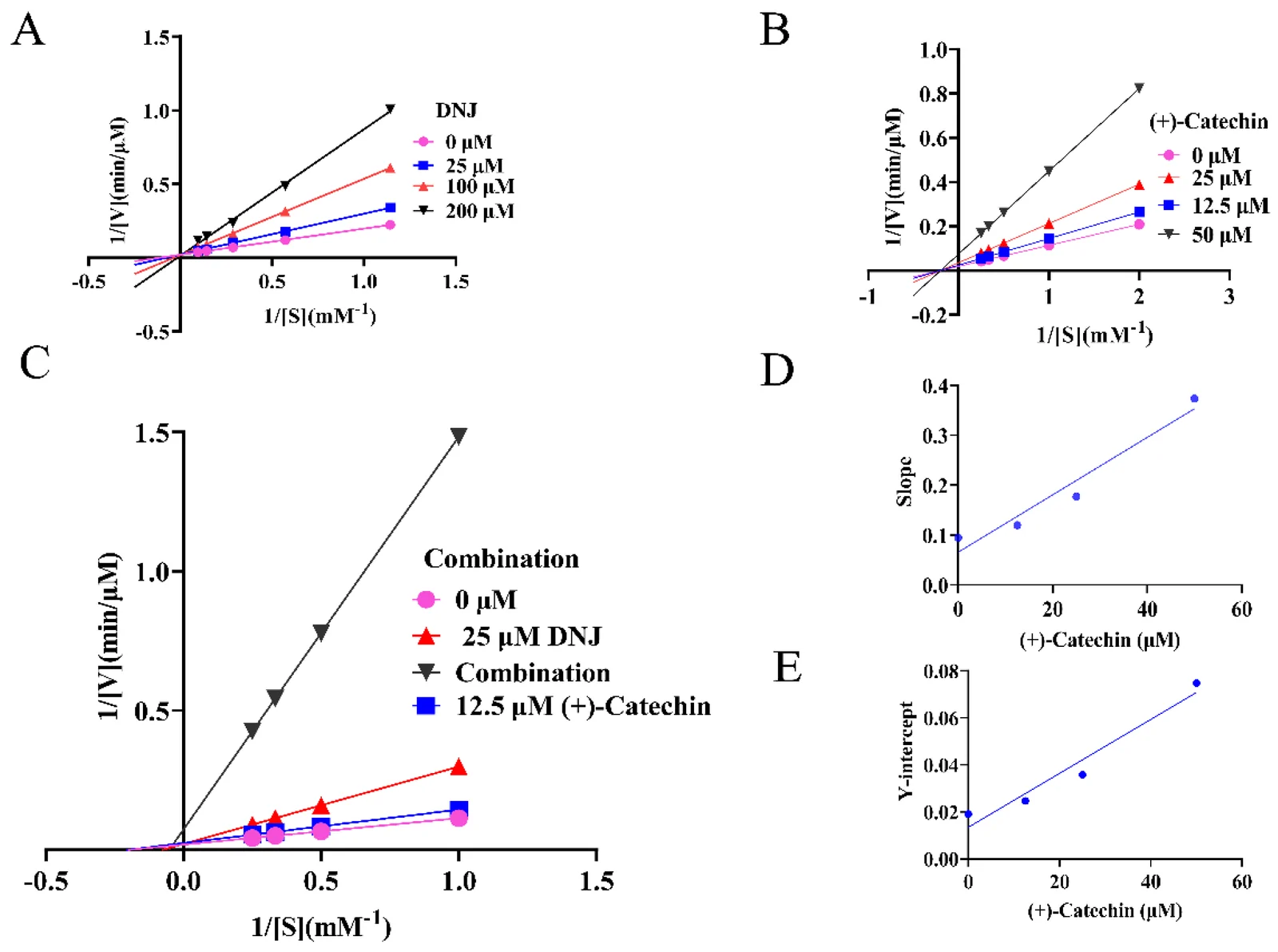
Kinetic analysis of DNJ, (+)-catechin, and their combination against α-glucosidase. (**A**–**C**) Lineweaver–Burk plots of substrate kinetics; (**D**) slope plot for (+)-catechin; (**E**) Y-intercept plot for (+)-catechin.

To clarify the synergistic mechanism of the combination composed of (+)-catechin and DNJ, the types of inhibition by (+)-catechin, DNJ, and their combination were determined by Lineweaver−Burk plots. As shown in Figure 4A, double-reciprocal plots for DNJ at different concentrations intersected on the y-axis. Increasing DNJ concentrations increased the *K*_m_ value, while *V*_max_ remained unchanged, indicating that DNJ acts as a competitive inhibitor; this is consistent with a previous report [23]. In contrast, plots for (+)-catechin intersected on the x-axis (Figure 4B) with *V*_max_ decreasing and *K*_m_ unchanged, characteristic of non-competitive inhibition. For the combined treatment of (+)-catechin and DNJ, the crossover point of the plots appeared in the second quadrant, corresponding to a mixed inhibition mode combining competitive and non-competitive characteristics (Figure 4C). Furthermore, the plot slope and y-intercept showed linear relationships with the (+)-catechin concentration, suggesting a single binding site for (+)-catechin on α-glucosidase (Figure 4D,E).

The enzyme kinetic parameters of α-glucosidase in the presence of DNJ, (+)-catechin, and their combination are summarized in Table 2. The inhibition constant *K*_i_ (11.4 μM) and the enzyme–substrate–inhibitor dissociation constant *K*_is_ (11.7 μM) for (+)-catechin were nearly identical, confirming comparable affinity for the free enzyme and the enzyme–substrate complex. In the combined treatment, the *K*_m_ value (19.2 μM) was higher than those of both DNJ (13.2 μM) alone and the control (5.0 μM), and *V*_max_ (13.6 μM/min) was lower than that of the control (52.5 μM/min). These distinct inhibition modes indicated that (+)-catechin and DNJ occupied different sites on α-glucosidase. As a non-competitive inhibitor, (+)-catechin alone did not alter substrate affinity; however, the combination further reduced substrate binding relative to DNJ alone, suggesting that (+)-catechin binding induced conformational changes that enhanced DNJ binding at the active site. This two-site mechanism is consistent with the synergistic inhibition reported by Dong et al. [24] for non-competitive 5,6,7-trihydroxy-flavonoid aglycones combined with DNJ, as well as with the findings of Abioye et al. [25] and Li et al. [26], who demonstrated that the non-competitive inhibitors chlorogenic acid and isoginkgetin, respectively, synergistically inhibited α-glucosidase when combined with the competitive inhibitor acarbose, with the combinations displaying mixed-type inhibition.

### 2.4. Fluorescence Quenching

**Figure 5:**
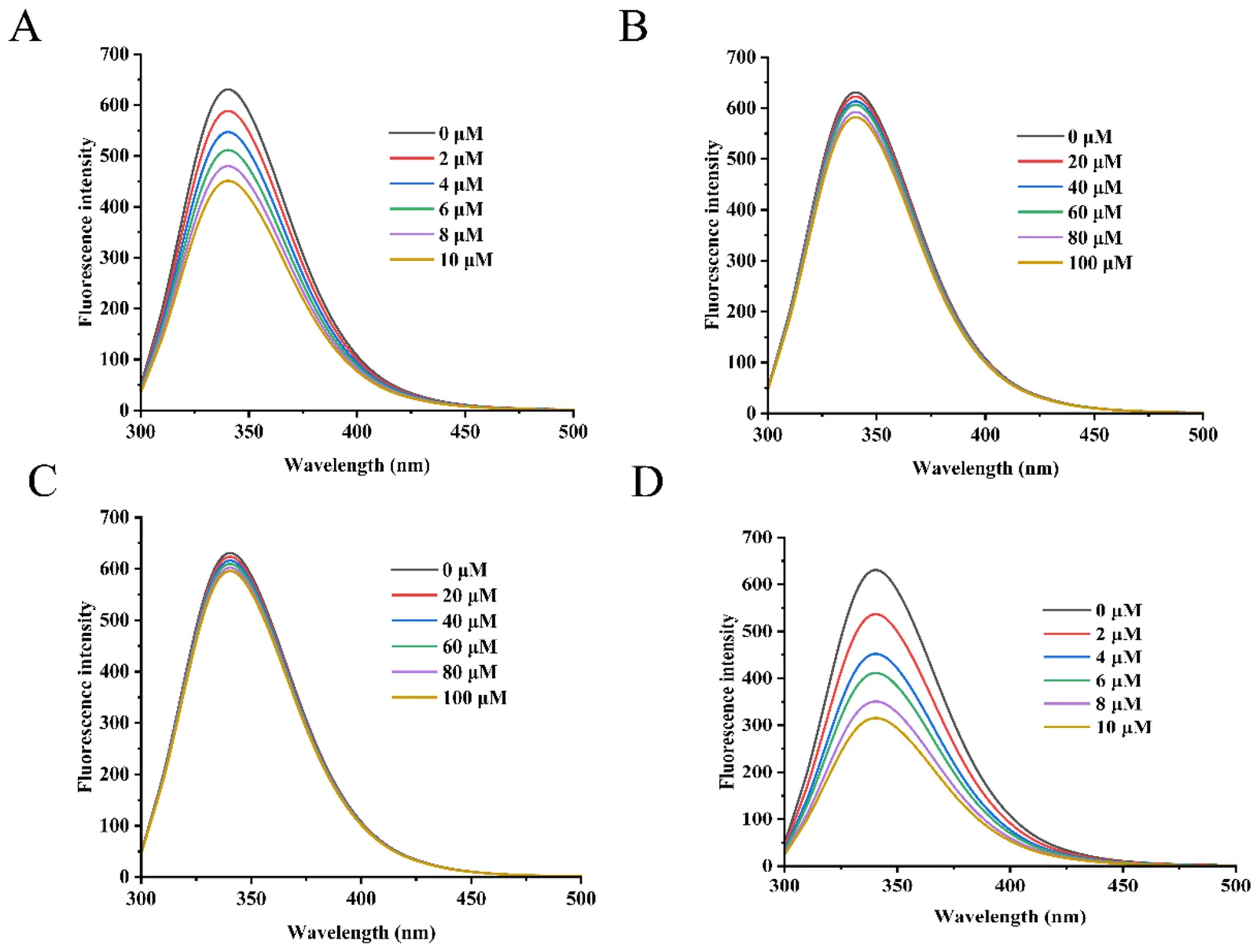
Fluorescence spectra of α-glucosidase under different treatment conditions: (**A**) (+)-catechin; (**B**) DNJ added to EC 1; (**C**) DNJ; (**D**) (+)-catechin added to EC 2. EC 1 refers to the enzyme complex pre-incubated with (+)-catechin; EC 2 refers to the enzyme complex pre-incubated with DNJ.

**Figure 6:**
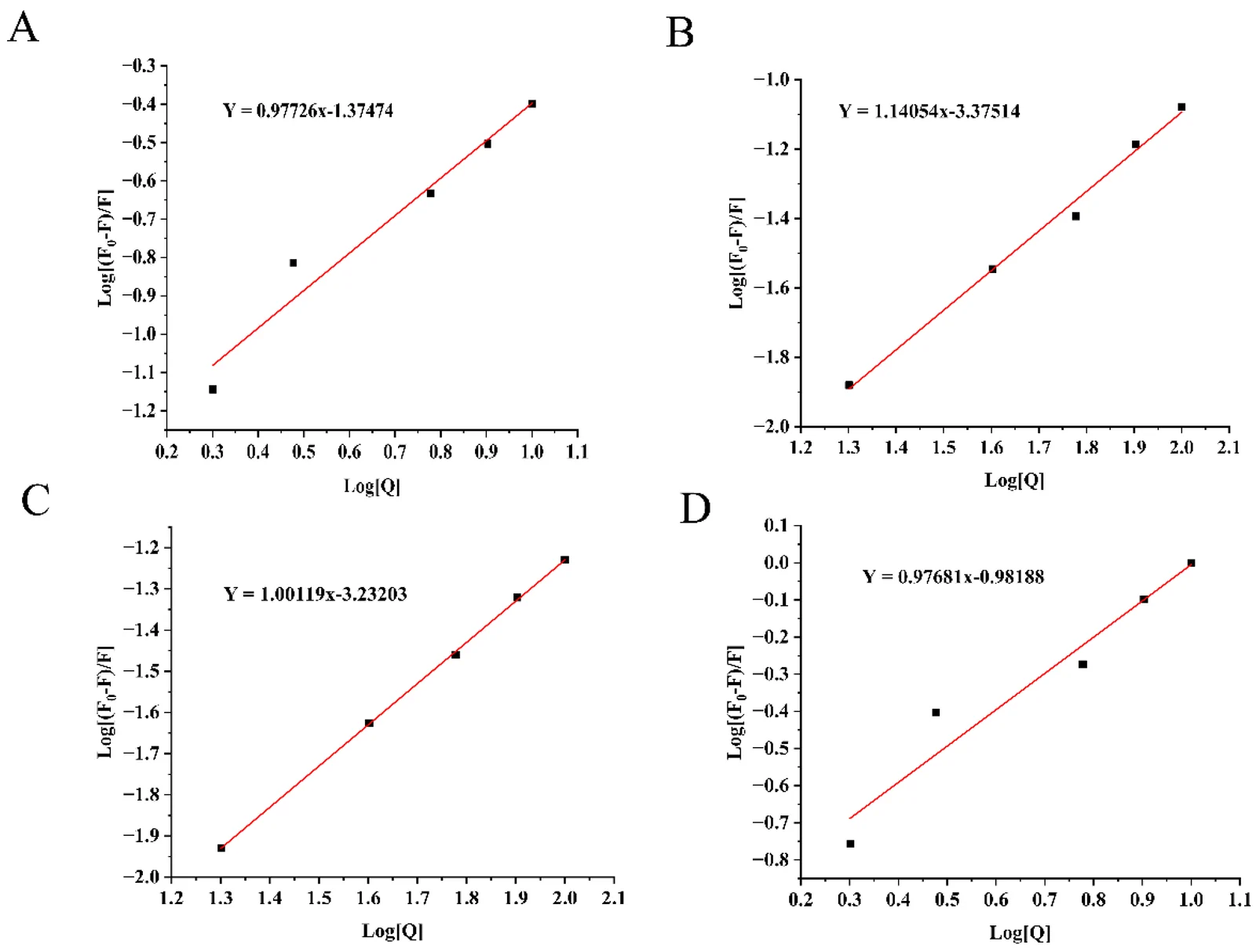
Plots of log [(*F*_0_ − *F*)/*F*] versus log[*Q*] for the interactions of DNJ, (+)-catechin, and their combination with α-glucosidase: (**A**) (+)-catechin; (**B**) DNJ added to EC 1; (**C**) DNJ; (**D**) (+)-catechin added to EC 2. EC 1 refers to the enzyme complex pre-incubated with (+)-catechin; EC 2 refers to the enzyme complex pre-incubated with DNJ.

To evaluate how the presence of one inhibitor affected the binding affinity of the other to α-glucosidase, the fluorescence spectra of α-glucosidase solutions treated with (+)-catechin, DNJ, and their sequential combinations were measured. The intrinsic fluorescence intensity of α-glucosidase decreased gradually with increasing inhibitor concentration, with no detectable shift in the emission peak (Figure 5), providing direct evidence for the interaction between the enzyme and both inhibitors. As shown in Figure 6, the plots of log [(*F*_0_ − *F*)/*F*] versus log[*Q*] showed good linearity. The calculated *n* values were approximately 1 for both (+)-catechin and DNJ, suggesting that each inhibitor occupied one binding site on α-glucosidase. Furthermore, the calculated *K*_q_ values (Table 3) exceeded the maximum collision quenching constant (2.0 × 10^10^ L·mol^−1^·s^−1^) [27], indicating that the fluorescence quenching proceeded exclusively via a static quenching mechanism involving the formation of non-fluorescent ground-state complexes.

Having established that quenching proceeded via a static mechanism, the binding constant (*K*_a_) and the number of binding sites (*n*) were determined for each inhibitor under the four experimental conditions (Table 3). The *K*_a_ (29.4 × 10^2^ L/mol) of DNJ binding to EC 1 ((+)-catechin–enzyme complex) was approximately five times that of DNJ alone (5.96 × 10^2^ L/mol), corresponding to a 393% increase in binding affinity. Similarly, the *K*_a_ (757 × 10^2^ L/mol) of (+)-catechin binding to EC 2 (DNJ–enzyme complex) exceeded that of (+)-catechin alone (308 × 10^2^ L/mol) by 145%. The calculated *n* values were approximately 1 for both sequential addition groups, confirming that each inhibitor occupies a single, distinct binding site on α-glucosidase. The asymmetry in the *K*_a_ enhancement, 393% for DNJ binding after (+)-catechin pre-incubation versus 145% for (+)-catechin binding after DNJ pre-incubation, indicated that (+)-catechin binding exerted a greater influence on the active site than DNJ binding exerted on the non-competitive site. This directionality suggested that (+)-catechin binding facilitated DNJ binding at the active site, rather than the two inhibitors mutually enhancing each other’s affinity to a comparable degree.

While fluorescence quenching is routinely applied to characterize single ligand–protein interactions, its use in assessing how pre-bound inhibitors mutually modulate each other’s binding affinity, as implemented here through the four sequential addition protocols, remains uncommon. This approach allowed direct quantification of the directionality and magnitude of affinity enhancement, providing experimental evidence. Combined with the enzyme kinetic results, these data demonstrated that (+)-catechin binding to a non-competitive site enhanced DNJ affinity for the active site and provided a molecular basis for the observed synergistic inhibition.

### 2.5. CD Spectroscopic Analysis

**Figure 7:**
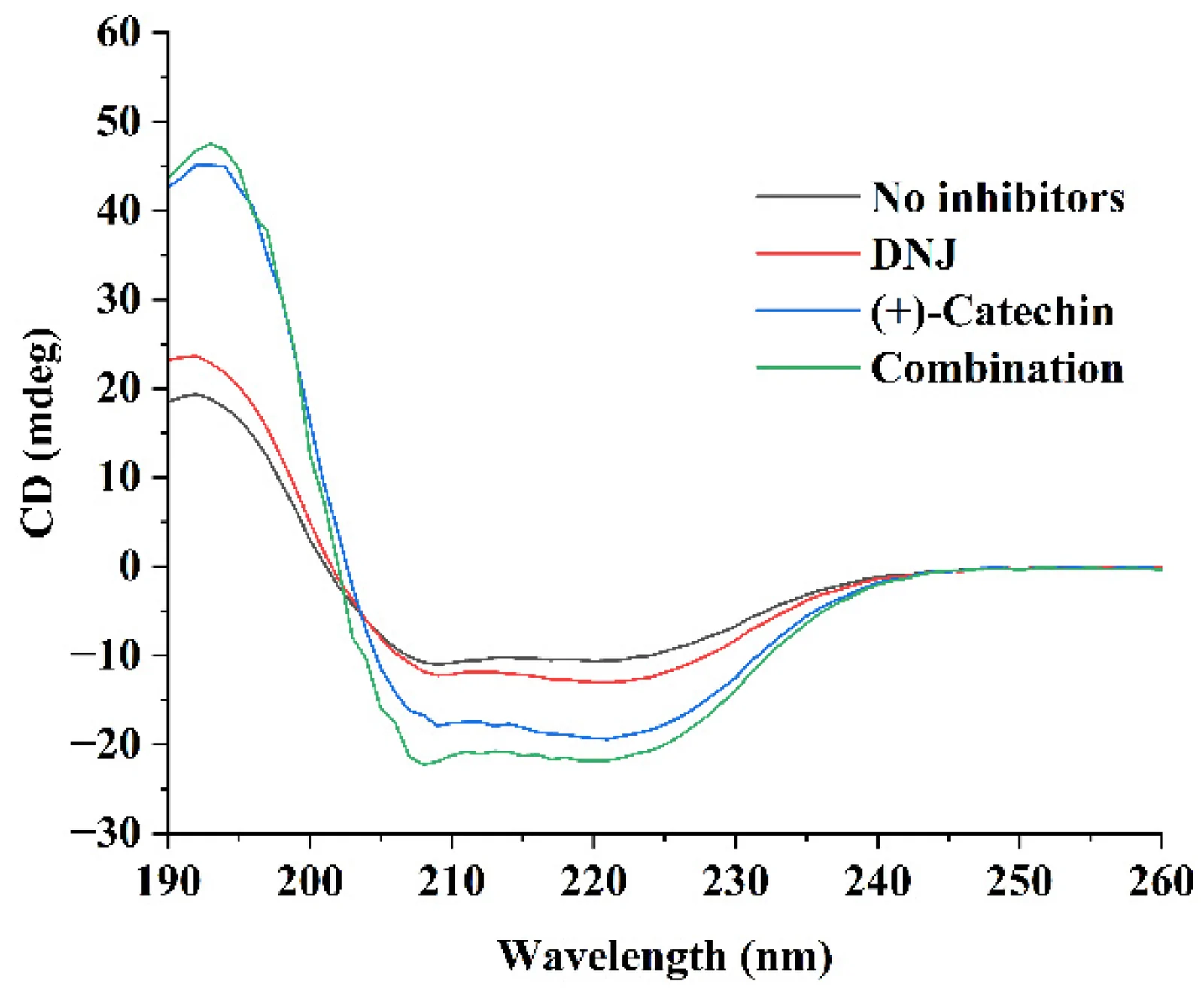
CD spectra of α-glucosidase with (+)-catechin and/or DNJ at pH 6.8 and room temperature. c(α-glucosidase) = 0.5 mg/mL, and the concentrations of (+)-catechin and DNJ were maintained at 0.01 mg/mL.

The fluorescence data implied that (+)-catechin binding induced conformational changes in α-glucosidase that enhanced its DNJ affinity. To test this hypothesis directly, CD spectroscopy was applied to characterize changes in the secondary structure of α-glucosidase under different treatments. As shown in Figure 7, the native enzyme exhibited a positive absorption band at 193 nm and two negative bands at 208 nm and 220 nm, characteristic of a mixed α-helix/β-sheet conformation. Quantitative spectral deconvolution using CDNN 2.1 (Table 4) showed that the secondary structure of free α-glucosidase consisted of 20.05% α-helices, 29.64% β-sheets, 16.65% β-turns and 33.74% random coils, consistent with previously reported CD results for α-glucosidase [28].

DNJ induced only minor structural alterations: the α-helix content increased slightly to 21.99%, the β-sheet content decreased to 26.84%, and random coils showed a modest rise. These data indicated that DNJ caused primarily local conformational adjustments rather than global structural changes, in agreement with He et al. [29]. In contrast, (+)-catechin triggered a marked conformational transition: the α-helix content rose substantially to 32.22%, the β-sheet content decreased sharply to 16.65%, and the β-turn and random coil proportions remained largely unchanged. This demonstrated that (+)-catechin binding strongly induced a β-sheet-to-α-helix conversion. Co-incubation with both inhibitors further increased the α-helix content to 35.76% and reduced the β-sheet content to 15.20%. These values exceeded the changes produced by either inhibitor alone, indicating that DNJ and (+)-catechin synergistically modified the secondary structure of α-glucosidase and thereby contributed to their combined inhibitory activity.

### 2.6. Molecular Docking

**Figure 8:**
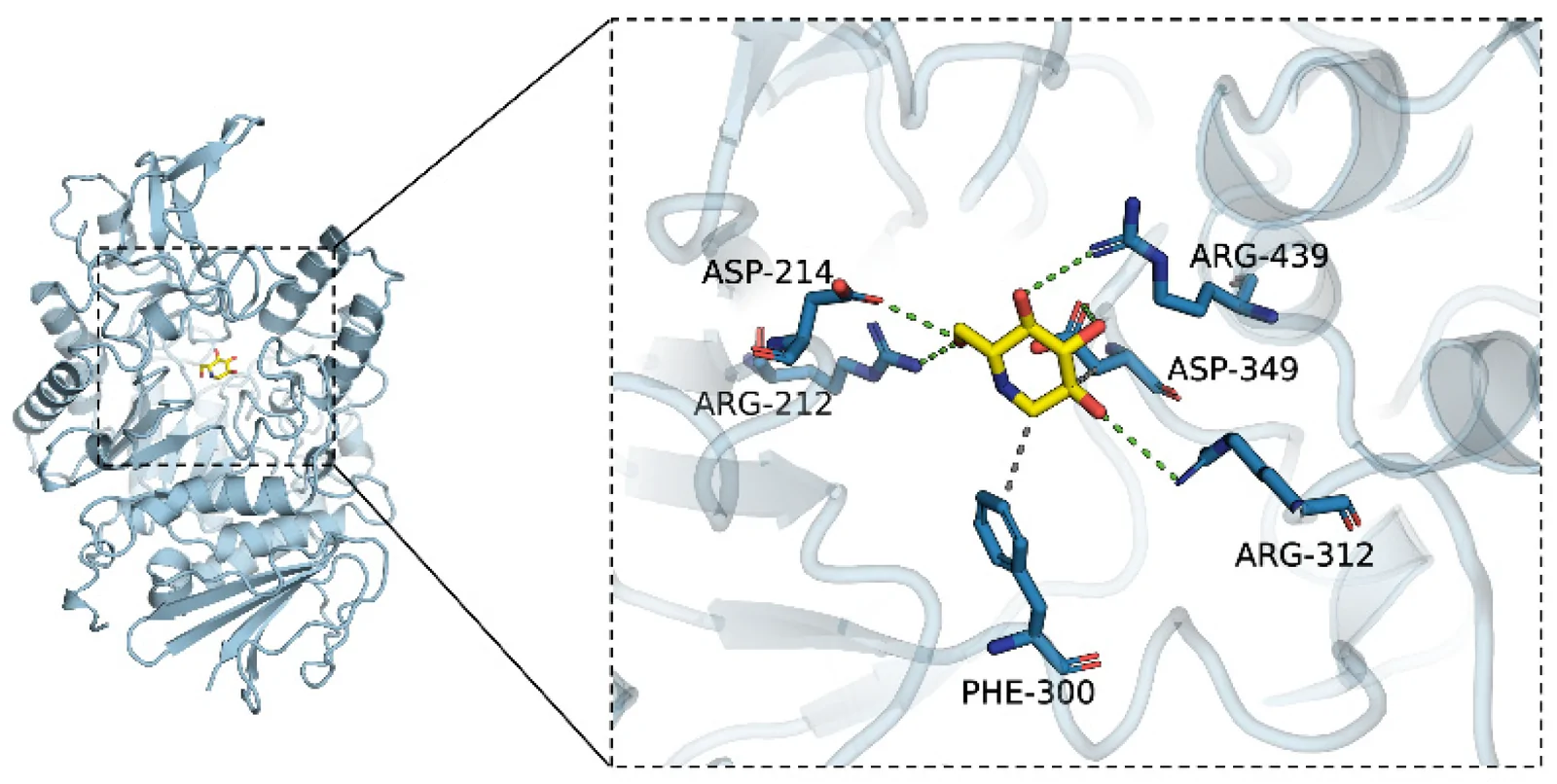
The binding mode of DNJ to α-glucosidase. DNJ is shown in yellow, surrounding residues in the binding pocket are shown in dark blue, and the receptor backbone is presented as a transparent white cartoon. Green dashed lines represent hydrogen bonds; gray dashed lines represent hydrophobic interactions.

To further clarify the binding modes of (+)-catechin and DNJ to α-glucosidase at the molecular level, molecular docking studies were performed. The docking score of (+)-catechin was −8.25 kcal/mol, more favorable than that of DNJ (−7.56 kcal/mol). As shown in Figure 8, DNJ binds to the active site of α-glucosidase, forming hydrophobic interactions with Asp349 and Phe300 and hydrogen bonds with Asp349, Arg439, Arg312, Asp214, and Arg212. Asp349 contributes to both hydrophobic interactions and the hydrogen bond network. This binding pattern, in which DNJ interacts with the conserved catalytic Asp349, is consistent with the canonical DNJ binding mode in GH13 α-glucosidases, as established by co-crystal structural data [30].

**Figure 9:**
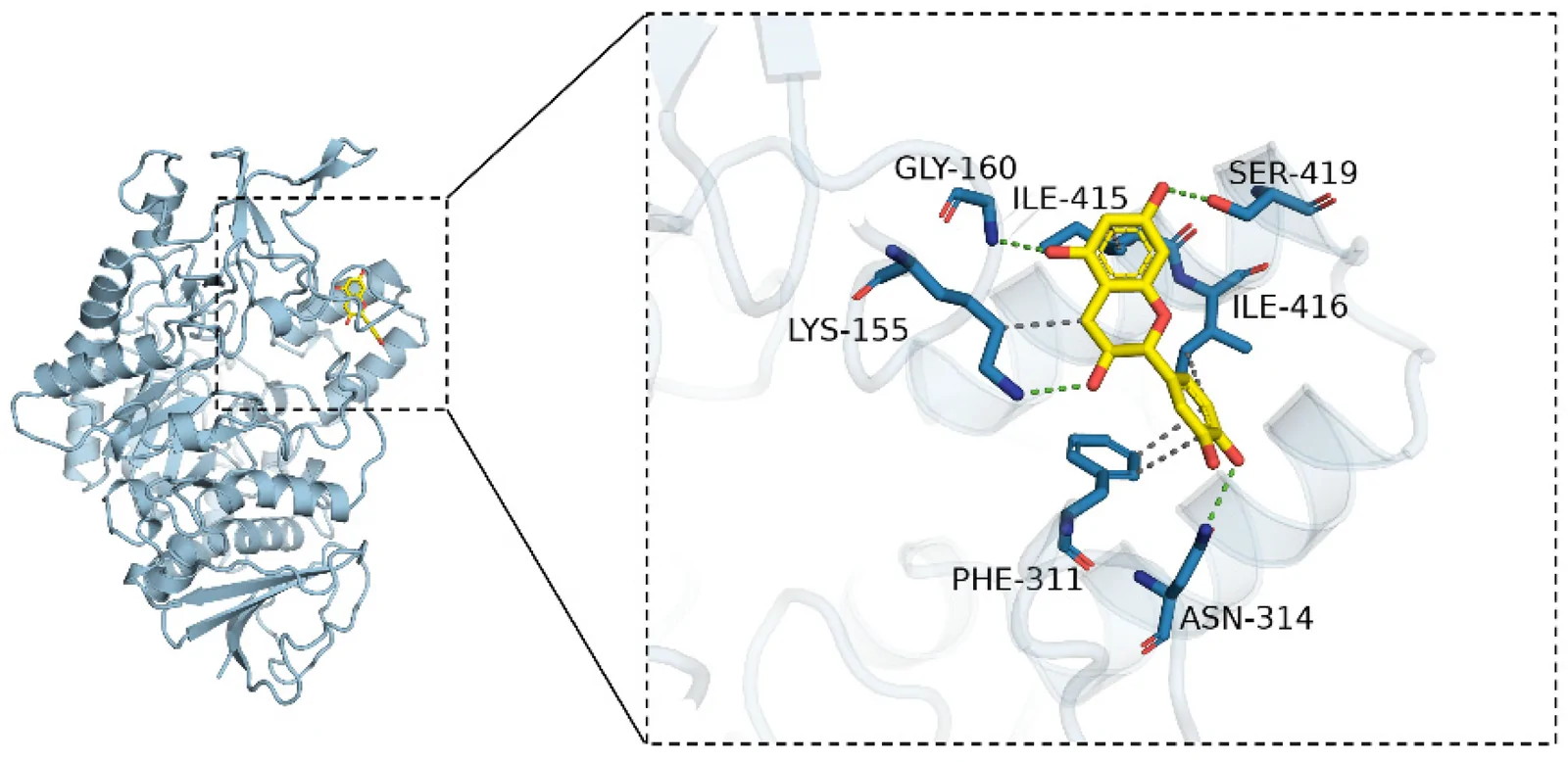
The binding mode of (+)-catechin to α-glucosidase. (+)-Catechin is shown in yellow, surrounding residues in the binding pocket are shown in dark blue, and the receptor backbone is presented as a transparent white cartoon. Green dashed lines represent hydrogen bonds; gray dashed lines represent hydrophobic interactions.

Figure 9 depicts the binding mode of (+)-catechin to α-glucosidase. The flavan core of (+)-catechin forms primary hydrophobic contacts with Phe311, Ile415, and Ile416, which, together with the aliphatic side chain of Lys155, establish a nonpolar microenvironment that accommodates the hydrophobic flavan skeleton of (+)-catechin within the binding pocket. In addition to hydrophobic interactions, the ligand forms hydrogen bonds with Ser419, Lys155, Asn314, and Gly160. Notably, Lys155 makes a dual contribution to complex stabilization: its aliphatic side chain participates in hydrophobic interactions, whereas its terminal charged amino group engages in hydrogen bonding. This dual interaction profile suggests that Lys155 is involved in both ligand recognition and conformational stabilization. The multiple hydrogen bonds further impose directional constraints on ligand positioning, thereby enhancing the overall stability of the (+) catechin–enzyme complex.

These docking results confirmed that DNJ and (+)-catechin occupy distinct binding sites: DNJ is bound at the catalytic active site, whereas (+)-catechin engages a separate allosteric pocket defined by Phe311, Ile415, Ile416, and Lys155. This two-site binding pattern is fully consistent with the distinct inhibition modes established by enzyme kinetics, the single non-overlapping binding sites indicated by fluorescence quenching, and the conformational changes detected by CD spectroscopy, providing atomic-level support for the proposed allosteric modulation mechanism.

## 3. Materials and Methods

### 3.1. Materials

a-Glucosidase from *Saccharomyces cerevisiae* (yeast) was purchased from Sigma-Aldrich (St. Louis, MO, USA). DNJ (purity ≥ 98%, verified by HPLC) and p-nitrophenyl-α-D-glucopyranoside (pNPG, purity ≥ 99%, verified by HPLC) were obtained from Macklin Biochemical Co., Ltd. (Shanghai, China). All flavonoid standards, (+)-catechin, quercetin, kaempferol, myricetin, apigenin, futeolin, fisetin, chrysin, and baicalein, were purchased from Yuanye Bio-Technology Co., Ltd. (Shanghai, China) (purity ≥ 97%, verified by HPLC). No compounds were isolated from plant material in this study. For enzymatic assays, stock solutions of DNJ and flavonoid standards were prepared by dissolving the compounds in a small volume of DMSO, followed by dilution to the required working concentrations with phosphate buffer (pH 6.8). The final DMSO concentration in all in vitro enzyme activity assay systems was maintained below 5% (*v*/*v*) to avoid interference with enzyme activity. All other reagents used in this study were of analytical grade and purchased from commercial suppliers.

### 3.2. α-Glucosidase Inhibitory Activity [31]

A 110 μL volume of 50 mM phosphate buffer (pH 6.8) was added to each well of a 96-well microplate, followed by the addition of 20 μL of 0.2 U/mL α-glucosidase solution and 10 μL of serially diluted inhibitor solutions. Each concentration was tested in triplicate wells per plate. The mixture was pre-incubated at 37 °C for 15 min, after which 20 μL of 2.5 mM pNPG substrate was added to initiate the enzymatic reaction. The plate was incubated for another 15 min at 37 °C, and 80 μL of 0.2 mol/L Na_2_CO_3_ solution was immediately added to terminate the reaction. The absorbance of the released 4-nitrophenol was read at 405 nm using a microplate reader. DNJ was used as a positive control. The inhibitor concentration required to inhibit 50% of the enzyme activity (IC_50_) was calculated via non-linear regression analysis using GraphPad Prism 8.0 software.

Inhibition% = (OD_control_ − OD_sample_)/OD_control_ × 100 (1)

Here, OD_control_ and OD_sample_ are the optical densities measured for the absorbance change without and with inhibitor, respectively.

### 3.3. Synergy Experiments

Based on the IC_50_ values, the combined inhibitory effect of each flavonoid paired with DNJ was investigated following a previously reported method with minor modifications [32]. Five concentration gradients of flavonoid and DNJ (1/4 IC_50_, 1/2 IC_50_, IC_50_, 2 IC_50_, 4 IC_50_) were designed for the combination experiments. The experimental procedure was identical to the single-compound α-glucosidase inhibition assay described above. The combination index (CI) values were calculated using CompuSyn software 1.4.

The CI calculation formula is as follows [33]:

CI = (D)_1_/(D_x_)_1_ + (D)_2_/(D_x_)_2_ (2)

where (D)_1_ and (D)_2_ represent the doses of the test flavonoid and DNJ required to reach a specific inhibition level in the combined system, respectively; (D_x_)_1_ and (D_x_)_2_ represent the doses of each single inhibitor that achieve the same inhibition level when used alone. CI values below 0.9, between 0.9 and 1.1, and above 1.1 correspond to synergistic, additive and antagonistic effects, respectively.

### 3.4. Inhibition Kinetic Assay

The inhibition types of (+)-catechin, DNJ, and their combination were determined by enzyme inhibition assays. Four pNPG substrate concentrations (0.25–10.5 mM) were used, together with (+)-catechin (0–50 μM), DNJ (0–200 μM), and a fixed combination (25 μM DNJ + 12.5 μM (+)-catechin). Inhibition modes were identified from Lineweaver–Burk plots, which are double-reciprocal plots of reaction rate against substrate concentration. The equations for the Lineweaver–Burk plot and secondary plots are as follows [34,35]:

1/*V* = *K*_m_/*V*_max_·1/[*S*] + 1/*V*_max_ (3)

*Slope* = *K*_m_/*V*_max_ + *K*_m_[*I*]/(*V*_max_*K_i_*) (4)

*Y-intercept* = 1/*V*_max_ + [*I*]/(*V*_max_*K*_is_) (5)

where *V* and *V*_max_ are the enzyme reaction’s initial rate and maximal velocity, respectively, in the absence and presence of inhibitors. *K*_i_, *K*_m_ and *K*_is_ are the inhibition constant, the Michaelis−Menten constant and the enzyme–substrate–inhibitor dissociation constant, respectively. [*I*] and [*S*] are the concentrations of the inhibitor and the substrate, respectively. The secondary plot slope was also determined. The values of *V*_max_ and *K*_m_ were calculated by intercepting the curves on the y- and x-axes, respectively. The values of *K*_i_ and *K*_is_ were sourced from Equations (4) and (5).

### 3.5. Fluorescence Spectra Experiment

Fluorescence spectra were recorded using a Hitachi F-7000 fluorescence spectrofluorometer (Hitachi, Tokyo, Japan). The measurement parameters were set as follows: excitation wavelength 280 nm; emission scan range 300–500 nm; excitation and emission slit widths 5 nm; PMT voltage 500 V; scan speed 1200 nm/min. PBS was used for baseline correction, and the intrinsic fluorescence of each inhibitor at the corresponding concentration was subtracted from the sample spectra.

Stock solutions of DNJ (1 mM), (+)-catechin (0.1 mM) and α-glucosidase (1 mg/mL) were freshly prepared with PBS before each experiment. Four fluorescence quenching assays were conducted: DNJ alone, (+)-catechin alone, sequential addition of DNJ to (+)-catechin-preincubated enzyme (DNJ to EC 1), and sequential addition of (+)-catechin to DNJ-preincubated enzyme ((+)-catechin to EC 2). The final reaction volume was 2 mL and contained 200 μL of 1 mg/mL α-glucosidase in a 1.0 cm path length quartz cuvette. For the individual inhibitor assays, the enzyme solution was mixed with DNJ (final concentration 0–100 μM) or (+)-catechin (final concentration 0–10 μM). For the DNJ to EC 1 assays, the enzyme solution was first mixed with 40 μL of 0.1 mM (+)-catechin, followed by DNJ at final concentrations of 0–100 μM. For the (+)-catechin to EC 2 assay, the enzyme solution was first mixed with 40 μL of 1 mM DNJ, followed by (+)-catechin at final concentrations of 0–10 μM. Samples were incubated at room temperature (298 K) for 30 min. The fluorescence emission intensity at 340 nm was used to calculate binding parameters.

To elucidate the quenching mechanism between the inhibitors and α-glucosidase, the Stern–Volmer quenching constant (*K*_SV_) was obtained by linear regression of *F*_0_/*F* versus [*Q*] (Equation (6)) [36], where *F*_0_ and *F* are the fluorescence intensities of α-glucosidase in the absence and presence of the quencher ((+)-catechin or DNJ), respectively, and [*Q*] is the quencher concentration. The binding constant *K*_a_ and the number of binding sites *n* were calculated via a double logarithmic equation by plotting *log*[(*F*_0_ − *F*)/*F*] against *log*[*Q*] (Equation (7)) [37].

*F*_0_/*F* = 1 + *K*_q_*τ*_0_[*Q*] = 1 + *K*_sv_[*Q*] (6)

*Log*[(*F*_0_ − *F*)/*F*] = *logK*_a_ + *nlog*[*Q*] (7)

### 3.6. CD Spectra

CD measurements were performed on a Chirascan V100 plus circular dichroism spectrometer (Bio-Logic MOS 450, BioLogic, Seyssinet-Pariset, France) at room temperature in the far-UV region (190–260 nm) under continuous nitrogen flow. The α-glucosidase concentration was fixed at 0.5 mg/mL in all test systems, and the concentrations of (+)-catechin and DNJ were both maintained at 0.01 mg/mL. The scanning speed was set to 100 nm/min with a bandwidth of 1 nm. All data are expressed as the mean residue ellipticity [θ] (mdeg). The CD spectrum of the buffer was subtracted for baseline correction. CD spectral data were processed with CDNN 2.1 to calculate the relative contents of different secondary structures of α-glucosidase.

### 3.7. Docking Research

Homology modeling was applied to construct the three-dimensional structure of yeast α-glucosidase MAL12 (UniProt accession: P53341), with the crystal structure of *Saccharomyces cerevisiae* oligo-1,6-glucosidase (PDB code: 3AXI; UniProt accession: P53051) adopted as the template, which shared 72.12% sequence identity with the target protein. The target protein crystal structures were downloaded from the Protein Data Bank (http://www.rcsb.org/) in PDB format. The protein structures were preprocessed using PyMOL 2.5.5 to remove water molecules and co-crystallized ligands. The three-dimensional structures of the compounds were retrieved from the PubChem database (https://pubchem.ncbi.nlm.nih.gov/) generated in pdbqt format. Molecular docking simulations were carried out using AutoDock Vina (version 1.2.6) [38], and all generated binding poses were scored and sorted via the program’s integrated native scoring function. The search grid box for docking was defined with its center at coordinates x = −21.828, y = −6.907, and z = −24.079 Å, with box dimensions adjusted separately to fit the molecular size of each respective ligand. The exhaustiveness value was fixed at 10, with all calculations deployed across 12 CPU threads. The receptor was maintained as rigid across the full docking process, whereas all ligands were granted full torsional flexibility. To verify the robustness of the optimized docking protocol, a redocking validation was performed using the co-crystallized ligand isolated from the 3AXI crystal structure. The root-mean-square deviation (RMSD) between the highest-ranked redocked conformation and the native crystallographic pose was calculated to be 1.075 Å, which lies well beneath the universally accepted 2.0 Å threshold for valid docking performance. This result verified that the parameter set applied in this study produces accurate and reproducible docking outcomes, confirming its appropriateness for follow-up binding mode investigations. Visualization of optimal docking conformations and in-depth analysis of ligand–receptor interaction patterns were performed with PyMOL 3.1 [39] coupled with Discovery Studio 2019.

## 4. Conclusions

In summary, this study systematically evaluated nine dietary flavonoids for their individual and DNJ-combined α-glucosidase inhibitory activities. (+)-Catechin, the only tested flavonoid lacking the C-ring 4-carbonyl group, exhibited the strongest individual inhibition (IC_50_ = 33.7 ± 2.7 μM) and was the only flavonoid that maintained synergistic inhibition with DNJ across all tested dose levels (CI < 0.7); all other flavonoids showed only moderate, dose-dependent synergy. Individual inhibitory potency did not predict synergistic behavior, indicating that the two properties are governed by distinct structural determinants.

Integrated evidence from enzyme kinetics, fluorescence quenching, CD spectroscopy, and molecular docking converged on an allosteric modulation mechanism. Enzyme kinetics established that DNJ acted as a competitive inhibitor at the catalytic active site, whereas (+)-catechin functioned as a non-competitive inhibitor at a distinct site. Fluorescence quenching further showed that (+)-catechin binding increased DNJ’s binding affinity by 393%, confirming that the two inhibitors occupy independent binding sites. CD spectroscopy revealed that (+)-catechin induced a β-sheet-to-α-helix conformational transition, providing the structural basis for this affinity enhancement. Molecular docking resolved these findings at the atomic level, showing that DNJ occupied the catalytic active site while (+)-catechin bound to a distinct allosteric pocket defined by Phe311, Ile415, Ile416, and Lys155.

These findings provide a mechanistic rationale for incorporating (+)-catechin into DNJ-containing nutraceutical formulations, potentially enabling effective α-glucosidase inhibition at reduced DNJ doses. However, this study was conducted exclusively in vitro using yeast-derived α-glucosidase. Therefore, it does not provide direct evidence for hypo- or hyperglycemic effects in vivo. Validation with mammalian intestinal α-glucosidase and in vivo models is required. In addition, the synergistic effects observed with pure analytical standards warrant further investigation in complex plant matrices, such as crude mulberry extracts, to assess whether similar synergy occurs under conditions relevant to practical product formulation. Importantly, we acknowledge that the proposed allosteric site and conformational changes are currently supported by enzyme kinetics, fluorescence quenching, CD spectroscopy, and molecular docking but should be further confirmed by direct structural or biophysical methods.

## Figures and Tables

**Table 1:** Individual inhibition of α-glucosidase by flavonoids.

Flavonoid	Inhibition (%) ^a^	IC_50_ (μM)
Baicalein	20.4 ± 7.8	>400
Chrysin	22.1 ± 8.9	>400
Apigenin	4.8 ± 2.3	>400
Luteolin	38.8 ± 2.1	>400
(+)-Catechin	82.7 ± 4.5	33.7 ± 2.7
Kaempferol	63.6 ± 4.1	163.2 ± 10.0
Quercetin	65.4 ± 3.4	160.0 ± 5.1
Myricetin	48.9 ± 3.3	395.0 ± 14.9
Fisetin	5.6 ± 2.2	>400
DNJ	76.4 ± 1.2	155.5 ± 17.2

^a^ Inhibition rate at 400 μM concentration for flavonoids and DNJ. All values are presented as mean ± standard deviation of triplicate assays.

**Table 2:** Effects of different concentrations of (+)-catechin, DNJ, and their combination on kinetic parameters of α-glucosidase using pNPG as substrate.

Concentration (μM)		*K*_m_ (μM)	*V*_max_ (μM/min)	*K*_i_ (μM)	*K*_is_ (μM)	Inhibition Mode
DNJ	0	8.4	47.6	54.5		Competitive
	25	13.2	47.6			
	100	27.5	52.6			
	200	44.7	52.2			
(+)-Catechin	0	5.0	52.6	11.4	11.7	Non-competitive
	12.5	4.9	40.7			
	25	4.9	27.9			
	50	5.0	13.4			
Combination	25:12.5	19.2	13.6			Mixed-type
Control	0	5.0	52.5			

**Table 3:** Quenching parameters of (+)-catechin and DNJ with α-glucosidase.

	*K*_sv_ (×10^2^ L/mol)	*K*_q_ (×10^10^ L/mol·s)	R^a^	*K*_a_ (×10^2^ L/mol)	*n*	R^b^
(+)-Catechin	390	390	0.987	308	0.98	0.948
DNJ to EC 1	8.35	8.35	0.983	29.4	1.14	0.990
DNJ	5.90	5.90	0.999	5.96	1.00	0.999
(+)-Catechin to EC 2	974	974	0.976	757	0.98	0.924

R^a^ represents the correlation coefficient of *K*_sv_ and *K*_q_ fitting; R^b^ represents the correlation coefficient of *K*_a_ fitting.

**Table 4:** Effects of (+)-catechin, DNJ, and their combination on α-glucosidase secondary structure.

	α-Helix	β-Sheet	Β-Turn	Random Coil
No inhibitors	20.05%	29.64%	16.65%	33.74%
DNJ	21.99%	26.84%	16.79%	34.38%
(+)-Catechin	32.22%	16.65%	16.45%	34.68%
Combination	35.76%	15.20%	16.72%	32.42%

## Data Availability

Data are contained within the article.
